# Seasonal changes of commercial traits, proximate and fatty acid compositions of the scallop *Flexopecten glaber* from the Mediterranean Sea (Southern Italy)

**DOI:** 10.7717/peerj.5810

**Published:** 2019-01-21

**Authors:** Ermelinda Prato, Francesca Biandolino, Isabella Parlapiano, Loredana Papa, Giuseppe Denti, Giovanni Fanelli

**Affiliations:** CNR-IAMC Istituto per l’Ambiente Marino Costiero, CNR-IRSA Water Research Institute, Taranto, Italy

**Keywords:** Scallop, Proximate composition, Fatty acids, Central Mediterranean Sea, Quality indeces

## Abstract

This study provides information on biological (gonadosomatic index), commercial quality (condition index and meat yield) and biochemical aspects (proximate composition, fatty acids) of the soft tissues of *Flexopecten glaber* reared in suspended cages in the Ionian Sea. The results showed that condition index (CI) and meat yield (MY) peaked in December (60 and 30%, respectively) and in April, May and June (from 53 to 60% for CI and from 34 to 36% for MY). Gonadosomatic index showed three main peaks in winter, spring and summer months. Contents of protein 8.18–11.9 g/100 g), lipid (0,.78–1.18 g/100 g) and carbohydrate (1.19–3.30 g/100 g) varied significantly during the study period. Saturated fatty acids was the dominant group, except in December when polyunsaturated fatty acids showed the highest proportion (43% of total FAs). Fatty acids of the n3 group were dominant with docosahexaenoic and eicosapentaenoic acids. Highest n3/n6 ratios were recorded in spring-summer specimens, with values > of 5. The results showed a better nutritional quality of scallops in May, July and December.

## Introduction

Bivalves are important marine organisms due to their nutritional, commercial and ecological relevance, appreciated for their taste, price, and their healthy attributes (Orban et al., 2002; Orban et al., 2006). Among them, scallops are considered a delicacy in many countries of the world. They represent an important part of the global seafood market and support both commercial fisheries and aquaculture all around the world (Shumway & Parsons, 2016). In 2014, world imports of scallops reached 157,200 t, with China as leading buyer (38,000 t) followed by the USA. The Italian market for scallops showed an average annual import at 5,800 t in the 2009–2014 period. The exploited European scallop species are *Pecten maximus*, *P. jacobaeus*, *Aequipecten opercularis*, *Flexopecten glaber* and *Mimachlamys varia*.

In Mediterranean coastal waters, *F. glaber*, is an endemic species, although fishing activity has severely reduced its distribution and abundance. The small quantities of scallops available via artisanal fisheries fail to meet the market demand (Turolla, 2008). Thus, to overcome such problems there is great interest in developing sustainable scallop culture, in line with the European Union strategy. The Italian shellfish market is based only on culture of two bivalve species: *Mytilus galloprovincialis* and *Ruditapes philippinarum*, while the rearing of other species of commercial interest (*Crassostrea gigas*, *Ostrea edulis, Ruditapes decussatus*) is limited (Prioli, 2004; Parisi et al., 2012). The lack of diversification, the organoleptic characteristics (taste, texture, firmness, etc.) and the high market value of *F. glaber*, comparable to that of the depleted wild Pilgrim’ scallop (*Pecten jacobeus*), make this species of great interest for the development of scallop culture (Marčeta et al., 2016). Prato et al. (2015), demonstrated that wild *F. glaber* spat in the Ionian waters is easy to collect on artificial substrates, where scallop juveniles have shown to be the most frequent and abundant of all settled bivalves. In addition, Tsiotisios et al. (2016) reported a high settlement density of *F. glaber* from the beginning of May to the end of August in the northwestern Amvrakikos Gulf (Ionian Sea, Greece).

The biochemical composition, together with the condition index, meat yield and gonadosomatic index are useful indicators of the nutritional and commercial quality of bivalves that may offer valuable information for decision-making processes on sustainable exploitation of this species (Orban et al., 2002; Orban et al., 2006). These parameters fluctuate as a result of the interaction between the variations in the seston (the natural diet of suspension-feeders) quality and quantity and the bivalves’ reproductive cycle (Pérez-Camacho et al., 2003; Orban et al., 2002; Orban et al., 2006; Park et al., 2011; Suárez et al., 2013; Baek et al., 2014).

Proteins, lipids, carbohydrates, free amino acids, vitamins, fatty acids, particularly the n3 polyunsaturated fatty acids (PUFAs) such as eicosapentaenoic (EPA, 20:5n3) and docosahexaenoic (DHA, 22:6n3) acids are major contributors to the nutritional value and organoleptic properties of molluscs (Orban et al., 2002; Orban et al., 2006).

Results of clinical and epidemiological researches have clearly shown the importance of EPA and DHA for human health (Chow, 2008). They play an important role in immune system regulation, prevention of cardiovascular disease and cancer, blood clots, neurotransmitters, cholesterol metabolism, and structure of membrane phospholipids in the brain and the retina, proper fetal development, anti-inflammatory properties and regulation of blood pressure (Simopoulos, 2002; Abedi & Ali Sahari, 2014).

As humans cannot synthesise essential PUFAs, they must be ingested with food (Kris-Etherton, Harris & Appel, 2002). These reasons justify the very high demand for this product category in national and international markets. Lipid and fatty acids composition of marine animals, including molluscs, are the most changeable components that are affected by season and capture areas (Prato et al., 2010; Prato & Biandolino, 2012).

Previous works on *F. glaber* reported on the occurence and distribution in the Mediterranean Sea (Peña et al., 1998; Le Pennec, Aloui-Bejaoui & Le Pennec, 2006; Dijkstra, 2013), its phylogeny (Pujolar et al., 2010), histopathological conditions, histological traits in the gonadic tissue during the reproductive cycle of the wild harvested adults from the North Western Adriatic Sea (Marčeta et al., 2016), the ultrastructural morphology of its spermatozoa (Le Pennec, Aloui-Bejaoui & Le Pennec, 2006) and the meat yield (Berik, Çankiriligil & Gül, 2017a). Although, some studies have reported on the proximate composition of *F. glaber*, few have paid attention to the fatty acids composition (Telahigue et al., 2010; Telahigue et al., 2013; Berik & Çankiriligil, 2013; Marčeta et al., 2016; Berik, Çankiriligil & Gül, 2017b).

The aim of this study was to provide new information regarding biological (gonadosomatic index), commercial quality (condition index and meat yield) and biochemical aspects (proximate composition, fatty acids) of *F. glaber* reared in suspended cages in the Gulf of Taranto (Ionain Sea). This study will provide useful information on this species, both to consumers about its quality during the different periods of the year and to farmers about its validity as a new and valuable economic resource along Ionian coasts (Central Mediterranean Sea).

## Material and Methods

### Samples collection and environmental conditions

Scallops were monthly collected (from September 2014 to August 2015) from cages suspended in the Gulf of Taranto, Ionian Sea (Mediterranean Sea: Latitude 40°25′54″N, Longitude 17°14′22″E). At each sampling time, scallops of commercial size (3.7–4.5 cm) were collected in triplicate (10 live scallops per replicate) and then immediately transported to the laboratory. Temperature, salinity and dissolved oxygen were measured monthly during the experimental period. Dissolved oxygen was measured in samples taken from a 13 m depth using a Niskin bottle, while water temperature and salinity were measured using a probe (IDROMAR IM 52).

### Condition index, meat yield and gonadosomatic index

After cleaning off epibenthic organisms, *F. glaber* was weighed to the nearest 0.01 g (W: whole wet weight) and the total length (LT: maximum length along the anterior–posterior axis) was measured using a stainless steel calliper (0.1 mm).

Following these measurements, condition index (CI) and meat yield (MY) were determined according to Orban et al. (2002) and Okumus & Stirling (1998).

\begin{eqnarray*}& & \text{CI}=[\text{meat wet weight (g)/shell wet weight (g)}]\times 100 \end{eqnarray*}

\begin{eqnarray*}& & \text{MY}=[\text{meat wet weight (g)/total wet weight (g)}]\times 100 \end{eqnarray*}

Gonads were removed and weighted for the determination of gonadosomatic index (GSI) calculated as:

\begin{eqnarray*}\text{GSI}=\text{Gonad wet weight(g) / Wet weight of meat (g)}\times 100. \end{eqnarray*}

### Proximate composition

Chemical analyses were performed on three samples of about 10 individuals each. All analyses were conducted in triplicate.

The moisture and ash contents into *F. glaber* were determined according to the standard procedures of AOAC (1995). Moisture content was calculated based on the percentage weight loss after drying to a constant weight at 105 °C overnight. Ash content was determined after having weighed and transferred the dry samples into a muffle furnace at 550 °C overnight.

The protein content was determined by the Bradford Protein Assay method (Bradford, 1976) with blue brilliant of Coomassie as the reagent. Standards were prepared from protein bovine serum albumin.

The chloroform-methanol lipid extraction and gravimetric determination of total lipid (TL) were performed following the method of Folch, Lees & Stanley (1957). The contents were expressed relative to the wet weight of the sample. Carbohydrates were quantified according to the phenol-sulphuric acid method using glucose as the standard (Dubois et al., 1956).

### Fatty acids analysis

The lipid extracts were subject to fatty acid analysis by gas chromatography after transmethylation into fatty acidmethyl esters (FAMEs). The methyl esters were prepared by adding a boron trifluoride-catalyzed methanol: benzene solution (1:2, v:v) to the lipid extract. The mixture was shaken vigorously, and then heated in boiling water for 45 min (Allinger et al., 1986). Samples were allowed to cool to room temperature, and 1 ml of distilled water was added followed by vigorous shaking. FAMEs were recovered in the upper phase, concentrated under nitrogen, and kept at −20 °C until further analysis. Triplicate samples were analysed.

Analysis of FAMEs was performed by gas chromatography (GC) using an HP 6890 series GC (Hewlett Packard, Wilmington, DE, USA) equipped with flame ionization detector. FAMEs were separated with an Omegawax 250 capillary column (Supelco, Bellafonte, PA, USA) (30 m long, 0.25-mm internal diameter, and 0.25-mm film thickness). Helium was used as the carrier gas at a flow rate of 1 ml/min. The column temperature program was as follows: 150 °C to 250 °C at 4 °C/min and then held at 250 °C. FAMEs were identified by comparing retention times with a standard (Supelco 37 Component FAME Mix). FAs were quantified by integrating areas under peaks in the GC traces, with calibration derived from an external standard containing different methyl esters. Relative quantities were expressed as weight % of total fatty acids. Percent of total fatty acids data were converted to amounts of mg/g wet soft tissue portion according to Greenfield & Southgate (2003).

### Lipid Nutritional Quality Indexes (LNQI)

Atherogenic index (AI), thrombogenicity index (TI) and hypocholesterolaemic/hypercholesterolaemic fatty acid ratio (HH) are commonly used to assess the nutritional value of lipid fraction and its potential effect on the development of coronary disease (Ulbricht & Southgate, 1991).

AI and TI were calculated according to the Ulbricht & Southgate (1991) equations:

\begin{eqnarray*}& & \text{AI}=(\text{C12:0}+4\times \text{C14:0}+\text{C16:0})/(\text{Sum MUFAs}+\text{Sum PUFAs}); \end{eqnarray*}

\begin{eqnarray*}\text{TI}& =& (\text{C14:0}+\text{C16:0}+\text{C18:0})/(0.5\times \text{Sum MUFAs}+0.5\times \text{Sum n6 PUFAs}\nonumber\\\displaystyle & & +3\times \text{Sum n3 PUFAs}+(\text{n3}/\text{n6})). \end{eqnarray*}

Where MUFAs are monounsaturated fatty acids and PUFAs are polyunsaturated fatty acids.

HH ratios was calculated according to the Fernández et al. (2007) equations:

\begin{eqnarray*}\text{HH}& =& (\text{C18}:\text{1cis9}+\text{C18:2n6}+\text{C20:4n6}+\text{C18:3n3}+\text{C20:5n3}\nonumber\\\displaystyle & & +\text{C22:5n3}+\text{C22:6n3})/(\text{C14:0}+\text{16:0}). \end{eqnarray*}

### Statistical analysis

Results are reported as means ± standard deviations (SD). Data were analysed for normality and variance homogeneity through Kolmogorov–Smirnov and Levene’s tests, respectively. Analysis of variance (ANOVA) was used to test significant seasonal differences in the commercial traits and fatty acid content among the analysed species. The multiple range test (Tukey’s test) was applied when the variance analysis indicated significant differences (*p* < 0.05). Pearson’s correlation analysis and *t*-test for significance of correlation, were applied to all environmental variables, lipid, protein and commercial data. PCA was performed on a data set of 12 cases (12 months) and 16 variables: ∑SAFA (sum of saturated fatty acid), ∑MUFA (sum of monounsaturated fatty acid), ∑PUFA (sum of polyunsaturated fatty acid) , n3 PUFA, n6 PUFA, n3/n6, n6/n3, PUFA/SAFA, UNS/SAT (unsaturated/saturated fatty acid), EPA (eicosapentaenoic fatty acid, 20:5n3), DHA (docosahexaenoic fatty acid, 22:6n3). DHA/EPA, DHA+EPA, AI, TI and HH. Statistics analyses were performed using XL-STAT (Version 2015.1) and STATISTICA software (Version 1.0) was used for principal components analysis (PCA).

## Results

**Figure 1:**
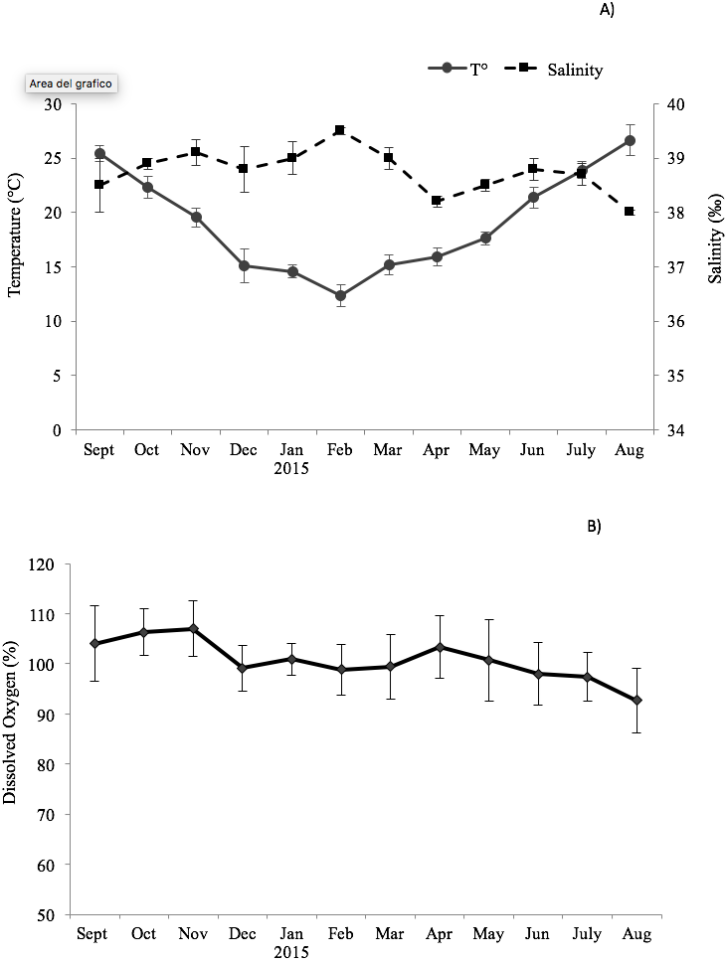
Trend of the environmental variables. Seasonal change of environmental variables measured at the sampling site in the Taranto Gulf from September 2014 to August 2015: (A) temperature (°C) and salinity (‰); (B) dissolved oxygen (%).

The environmental variables recorded monthly are shown in Fig. 1. The seawater temperature fluctuated between a minimum of 14 °C in February and March to a maximum of 26.7 °C in August. In the same way, dissolved oxygen showed significant seasonal changes with maximum values in autumn months (104–107%) and minimum in summer months (92.7–98%). The salinity did not display significant changes, remaining almost constant in the study period (39–38.2‰).

**Figure 2:**
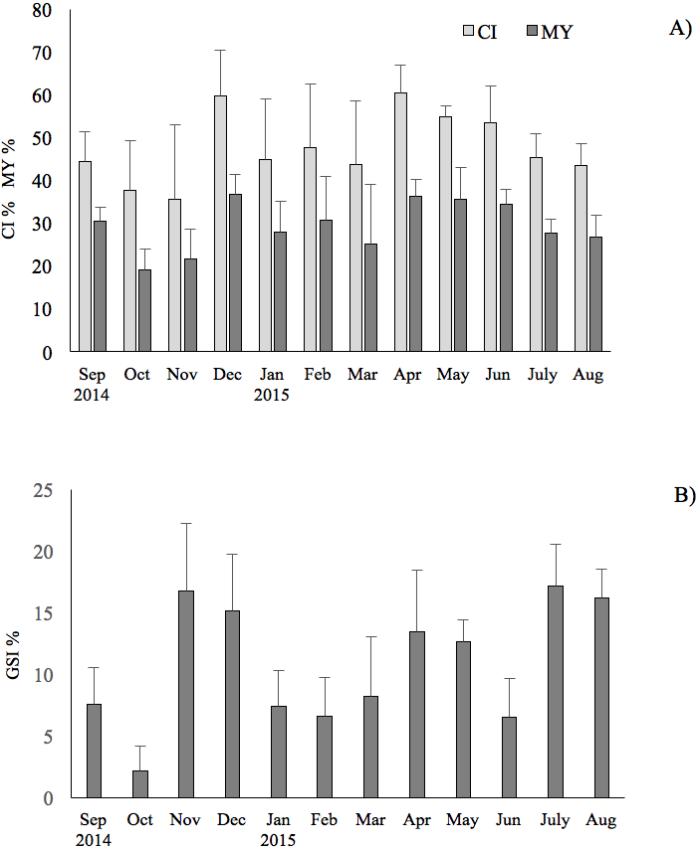
Seasonal changes of condition index, meat yield and gonadosomatic index of the *F. glaber*. Monthly changes of condition index (CI), meat yield (MY) (A) and gonadosomatic index (B) of *Flexopecten glaber* reared in the Gulf of Taranto from September 2014 to August 2015. Standard deviations are indicated by vertical black lines.

The results showed that the commercial traits CI and MY of *F. glaber*, reared in suspended cages varied at different sampling times of the year. They showed very similar patterns (*r* = 0.93, *p* < 0.05), with peak of values in December 2014, in April and May 2015, whereas lower values were found in October-November (ANOVA, *p* < 0.05) (Fig. 2).

GSI exhibited three evident peaks throughout the study period, the first one in July (17%) through August (16% ), the second one in November (16.7%)–December (15%) and the latest in April (13.4%) and in May (12.6%). The results obtained showed a very low correlation of GSI with CI and MY (*r* = 0.20 and 0.21, respectively; *t*-test: *p* < 0.05) during the study period.

The proximate composition of smooth scallop is shown in Table 1. The moisture percent was highest in March (84.81%), while ash content ranged between 2.27% in March and 2.87% in July.

**Table 1:** Seasonal changes of proximate composition of *Flexopecten glaber* during the study period. Monthly changes in moisture, ash, protein, lipid and carbohydrate of the edible portion of *Flexopecten glaber* reared in the Gulf of Taranto from September 2014 to August 2015. Values are mean ± S.D. (*N* = 30, *n* = 3). Data with different superscript letters differ significantly, *p* < 0.05.

	Sep	Oct	Nov	Dec	Jan	Feb
Moisture (g/100 g)	82.67 ± 1.13^a^	82.56 ± 1.14^a^	83.23 ± 0.62^a^	82.53 ± 0.73^a^	82.51 ± 1.07^a^	82.14 ± 0.95^a^
Ash (g/100 g)	2.45 ± 0.13^a^	2.65 ± 0.09^b,c^	2.74 ± 0.12^b^	2.51 ± 0.09^a^	2.64 ± 0.14^b,c^	2.68 ± 0.15^b,c^
Protein (g/100 g)	11.29 ± 0.95^c,d,e^	8.99 ± 0.74^a,b^	9.77 ± 0.64^a,b,c,d^	10.71 ± 1.04 ^b,c,d,e^	10.26 ± 0.94^b,c,d,e^	9.39 ± 0.84^a,b,c^
Lipid (g/100 g)	1.21 ± 0.10^f^	0.86 ± 0.11^a,b,c^	0.82 ± 0.09^a,b^	0.98 ± 0.11^ b,c,d,e^	0.88 ± 0.09^a,b,c,d^	0.84 ± 0.06^a,b^
Carbohydrate (g/100 g)	1.24 ± 0.07^a^	3.30 ± 0.15^f^	2.47 ± 0.11^c,d^	2.46 ± 0.09^c,d^	2.63 ± 0.11^d^	3.07 ± 0.18^e^
	**Mar**	**Apr**	**May**	**Jun**	**July**	**Aug**
Moisture %	84.81 ± 1.57^b^	82.65 ± 0.44^a^	82.41 ± 1.27^a^	82.28 ± 1.67^a^	82.17 ± 1.14^a^	82.37 ± 1.34^a^
Ash %	2.27 ± 0.11^d^	2.53 ± 0.10^a^	2.45 ± 0.09^a^	2.42 ± 0.14^a^	2.87 ± 0.16^c^	2.67 ± 0.10^b,c^
Protein (g/100 g)	8.18 ± 0.45^a^	10.64 ± 0.40^b,c,d,e^	10.81 ± 1.07^b,c,d,e^	11.57 ± 1.13^d,e^	11.99 ± 1.27^e^	11.68 ± 1.17^d,e^
Lipid (g/100 g)	0.78 ± 0.08^a^	1.05 ± 0.12^c,d,e,f^	1.14 ± 0.08^e,f^	1.07 ± 0.09^d,e,f^	1.16 ± 0.07^e,f^	1.18 ± 0.09^f^
Carbohydrate (g/100 g)	2.89 ± 0.12^e^	2.26 ± 0.09^c^	1.78 ± 0.08^b^	1.65 ± 0.11^b^	1.19 ± 0.09^a^	1.27 ± 0.04^a^

After moisture, protein was the main component of *F. glaber*, accounting for 8.18 g/100 g ww in March to 11.99 g/100 g ww in July (Table 1). Lipid ranged from a minimum value of 0.78 g/100 g ww in March to maximum values in July–September (1.16–1.21 g/100 g ww, respectively). Both, protein and lipid showed a similar pattern with highest values during spring-summer and the lowest in autumn-winter months (*r* = 0.89; *t*-test, *p* < 0.05); moreover, a positive correlation was found with the seawater temperature (*r* = 0.70, *r* = 0.64, for lipid and protein, respectively; *t*-test, *p* < 0.05).

Carbohydrate content showed an opposite trend to that of the protein and lipid (*r* =  − 0.89; *t*-test, *p* < 0.05) (Table 1).

A detailed fatty acids profile of the lipid extracted from *F. glaber* is shown in Table 2.

**Table 2:** Fatty acids composition (mg /100 g) of *Flexopecten glaber* reared in the Gulf of Taranto from September 2014 to August 2015. In the bracket are reported the % values of each Fatty acids. Values are mean ± S.D. (*N* = 30, *n* = 3). The sum of saturated, monounsaturated and polyunsaturated fatty acids (mean ± sd) are reported in bold. Different letters indicate significant differences for data in the same row (*p* < 0.05).

	September	October	November	December	January	February
C12:0	6.09 ± 1.19 (0.70)	11.13 ± 0.84 (1.80)	10.22 ± 2.33 (1.78)	5.50 ± 0.05 (0.80)	5.90 ± 1.44 (0.96)	6.47 ± 0.25 (1.10)
C14:0	10.49 ± 2.75 (1.20)	57.71 ± 0.77 (9.60)	80.20 ± 10.43 (13.97)	18.12 ± 3.43 (2.64)	48.59 ± 7.44 (7.89)	46.61 ± 6.72 (7.93)
C15:0		0.20 ± 0.08 (0.10)				
C16:0	289.32 ± 16.0 (34.20)	161.57 ± 3.57 (26.80)	209.43 ± 1.39 (36.49)	173.40 ± 18.21 (25.28)	181.11 ± 0.97 (29.40)	170.43 ± 4.46 (28.98)
C17:0	24.53 ± 0.71(2.90)	7.21 ± 0.55 (1.20)	9.64 ± 0.27 (1.68)	9.61 ± 1.11 (1.40)	8.02 ± 0.06 (1.30)	9.30 ± 0.08 (1.58)
C18:0	94.68 ± 5.69 (11.20)	25.88 ± 0.10 (4.30)	38.0 ± 5.73 (6.62)	40.06 ± 0.15 (5.84)	29.15 ± 0.72 (4.73)	41.96 ± 5.03 (7.14)
C20:0		0.74 ± 0.08 (0.10)	0.62 ± 0.33 (0.11)			
C21:0	2.89 ± 0.41(0.30)	0.97 ± 0.09 (0.20)	0.69 ± 0.19 (0.12)	2.27 ± 0.13 (0.33)	1.27 ± 0.07 (0.21)	0.82 ± 0.29 (0.14 )
∑SAFA	**428.01 ± 25.94**^**a**^**(50.50)**	**265.42 ± 2.91**^**f,g**^**(44.10)**	**348.82 ± 11.62**^**c,d**^**(60.77)**	**248.95 ± 22.71**^**g**^**(36.29)**	**274.04 ± 10.45**^**f**^**(44.49)**	**275.59 ± 6.03**^**f**^**(46.87)**
C14:1	5.76 ± 0.05 (0.68)	2.23 ± 0.48 (0.37)	0.86 ± 0.33 (0.15)	0.73 ± 0.18 (0.11)	1.53 ± 0.00 (0.25)	0.80 ± 0.26 (0.14)
C15:1	2.98 ± 1.13 (0.36)			3.54 ± 1.22 (0.52)		
C16:1	48.38 ± 3.15 (5.71)	111.72 ± 3.74 (18.56)	70.62 ± 2.91 (12.30)	60.43 ± 9.40 (8.81)	73.15 ± 2.03 (11.87)	46.32 ± 3.58 (7.88)
C17:1	8.22 ± 0.02 (0.97)	4.35 ± 0.53 (0.72)	4.02 ± 0.35 (0.70)	5.38 ± 0.75 (0.78)	5.45 ± 0.30 (0.95)	4.70 ± 0.26 (0.80)
C18:1n7	4.92 ± 1.26 (0.58)	11.04 ± 3.79 (1.83)	24.88 ± 6.30 (4.33)	19.01 ± 0.16 (2.77)	16.25 ± 0.40 (2.64)	14.54 ± 0.78 (2.47)
C18:1n9t						4.69 ± 1.32 (0.80)
C18:1n9c	100.57 ± 11.08 (11.87)	36.24 ± 6.98 (6.02)	37.88 ± 5.61 (6.60)	50.59 ± 4.70 (7.37)	43.00 ± 1.12 (6.98)	43.76 ± 1.38 (7.44)
C20:1n9	3.19 ± 1.02 (0.38)	0.74 ± 0.05 (0.12)	3.40 ± 0.14 (0.59)			
C22:1n9	1.70 ± 0.26 (0.20)	1.87 ± 0.23 (0.31)	0.63 ± 0.05 (0.11)	3.57 ± 1.48 (0.52)	1.47 ± 0.14 (0.24)	2.32 ± 0.71 (0.39)
∑MUFA	**175.72 ± 14.95**^**a**^**(20.76)**	**168.20 ± 6.79**^**a,b**^**(27.94)**	**142.29 ± 3.02**^**d**^**(24.79)**	**143.26 ± 11.98**^**d**^**(20.88)**	**141.25 ± 3.99**^**d**^**(22.93)**	**117.13 ± 0.93**^**e**^**(19.92)**
C18:2n6t		1.12 ± 0.38 (0.19)	2.74 ± 0.91 (0.48)			
C18:2n6c	22.94 ± 1.24 (2.71)	11.89 ± 0.51 (1.98)	8.73 ± 1.58 (1.52)	17.16 ± 0.51 (2.50)	15.70 ± 0.45 (2.55)	15.81 ± 0.41 (2.69)
C18:3n6	2.76 ± 0.25 (0.33)	2.87 ± 0.15 (0.47)	0.69 ± 0.21 (0.12)	3.39 ± 0.35 (0.49)	2.08 ± 0.09 (0.34)	1.88 ± 0.01 (0.32)
C18:3n3	25.12 ± 5.21 (2.97)	10.01 ± 0.23 (1.66)	7.77 ± 1.19 (1.35)	21.66 ± 0.42 (3.16)	18.29 ± 0.68 (2.97)	17.95 ± 0.00 (3.05)
C18:4n3	17.61 ± 3.55 (2.08)	16.15 ± 0.68 (2.69)	5.09 ± 1.21 (0.89)	37.03 ± 2.85 (5.39)	41.91 ± 1.74 (6.80)	39.44 ± 1.27 (6.71)
C20:2	1.46 ± 0.32 (0.17)	0.42 ± 0.05 (0.07)	0.51 ± 0.17 (0.09)	3.68 ± 1.48 (0.54)		
C22:0 + 20:3n6	1.54 ± 0.54 (0.18)	1.44 ± 0.01 (0.24)	0.46 ± 0.15 (0.08)	6.48 ± 2.59(0.95)	0.89 ± 0.00 (0.14)	0.55 ± 0.08 (0.09)
C20:3n3 + 22:1	1.35 ± 0.56 (0.16)	0.36 ± 0.00 (0.06)		7.89 ± 3.36 (1.15)		0.60 ± 0.01 (0.10)
C20:4n6	27.10 ± 2.54 (3.20)	16.96 ± 0.53 (2.82)	9.15 ± 1.08 (1.59)	26.98 ± 6.76 (3.93)	14.34 ± 0.28 (2.33)	15.83 ± 0.62 (2.69)
C22:2	1.22 ± 0.22 (0.14)	0.54 ± 0.09 (0.09)	1.84 ± 0.43 (0.32)			
C20:5n3	57.89 ± 9.61^e,f^(6.83)	67.71 ± 2.40^c,d^(11.23)	26.24 ± 2.87^g^ (4.57)	94.36 ± 13.21^a^(13.75)	64.29 ± 1.92^d,c^ (10.44)	55.0 ± 1.77^f^(9.35)
C22:5n3	2.83 ± 1.03 (0.33)	2.05 ± 1.03 (0.35)		2.95 ± 1.22 (0.43)	1.20 ± 0.06 (0.19)	2.19 ± 0.78 (0.37)
C22:6n3	81.39 ± 12.86^b,c^ (9.60)	36.85 ± 1.80^f^ (6.10)	19.65 ± 1.12^g^(3.42)	72.21 ± 5.12^c,d^(10.52)	42.01 ± 1.23^f^(6.82)	46.02 ± 4.72^e,f^(7.82)
∑ PUFA	**243.22 ± 40.84**^**c**^**(28.71)**	**168.37 ± 3.88**^**e**^**(27.97)**	**82.89 ± 8.60**^**f**^**(14.44)**	**293.78 ± 5.67**^**a**^**(42.83)**	**200.71 ± 6.45**^**d**^**(32.58)**	**195.28 ± 6.97**^**d,e**^**(33.21)**

	March	April	May	June	July	August
C12:0	2.76 ± 0.19 (0.51)	3.40 ± 0.47 (0.46)	2.96 ± 0.26 (0.37)	3.02 ± 0.30 (0.40)	3.09 ± 0.64 (0.38)	3.72 ± 0.51 (0.45)
C14:0	30.38 ± 5.47 (5.56)	38.77 ± 6.48 (5.27)	54.68 ± 1.39 (6.85)	59.27 ± 14.63 (7.91)	22.20 ± 3.04 (2.73)	35.26 ± 3.39 (4.27)
C15:0				0.44 ± 0.06 (0.06)		
C16:0	163.94 ± 6.87 (30.02)	204.78 ± 0.24 (27.86)	214.17 ± 6.24 (26.84)	206.76 ± 3.41 (27.60)	247.81 ± 8.75 (30.52)	257.95 ± 10.14 (31.23)
C17:0	9.04 ± 0.92 (1.65)	12.13 ± 0.14 (1.65)	13.67 ± 0.40 (1.71)	13.27 ± 2.41 (1.77)	17.15 ± 0.60 (2.11)	19.49 ± 0.59 (2.36)
C18:0	34.21 ± 3.37 (6.27)	40.28 ± 0.99 (5.48)	45.01 ± 2.33 (5.64)	42.86 ± 9.88 (5.72)	56.94 ± 1.22 (7.01)	67.01 ± 2.89 (8.11)
C20:0						
C21:0	1.48 ± 0.18 (0.27)	2.28 ± 0.00 (0.31)	4.07 ± 0.34 (0.51)	3.32 ± 0.04 (0.44)	4.26 ± 1.73 (0.52)	4.49 ± 0.30 (0.54)
∑SAFA	**241.82 ± 7.68**^**h**^**(44.29)**	**301.66 ± 6.35**^**e**^**(41.04)**	**334.56 ± 5.63**^**c,d**^**(41.92)**	**328.95 ± 6.06**^**d**^**(43.92)**	**351.45 ± 11.31**^**c**^**(43.28)**	**387.91 ± 9.27**^**b**^**(46.96)**
C14:1	1.16 ± 0.10 (0.21)	1.40 ± 0.13 (0.19)			1.81 ± 0.13 (0.22)	
C15:1						
C16:1	46.22 ± 3.69 (8.46)	68.33 ± 0.83 (9.30)	68.18 ± 1.49 (8.57)	64.32 ± 7.53 (8.59)	69.10 ± 3.63 (8.51)	59.89 ± 1.05 (7.25)
C17:1	5.04 ± 0.16 (0.92)	7.03 ± 0.04 (0.96)	6.98 ± 0.43 (0.87)	7.25 ± 0.28 (0.97)	7.27 ± 1.00 (0.89)	7.57 ± 0.45 (0.92)
C18:1n7	12.65 ± 0.69 (2.32)	11.18 ± 6.82 (1.52)	10.97 ± 0.50 (1.37)	9.11 ± 5.75 (1.22)	1.85 ± 0.20 (0.23)	9.27 ± 0.53 (1.12)
C18:1n9t	3.15 ± 0.20 (0.58)	3.98 ± 0.11 (0.54)	5.94 ± 0.30 (0.74)			5.58 ± 0.08 (0.67)
C18:1n9c	40.47 ± 1.96 (7.41)	56.27 ± 10.46 (7.66)	63.73 ± 0.52 (7.98)	60.83 ± 4.62 (8.12)	74.67 ± 2.89 (9.20)	84.71 ± 4.84 (10.26)
C20:1n9	0.34 ± 0.10 (0.06)	2.77 ± 0.04 (0.38)	2.99 ± 0.05 (0.37)	1.77 ± 0.63 (0.24)	2.12 ± 1.08 (0.26)	2.87 ± 0.12 (0.35)
C22:1n9	1.98 ± 0.19 (0.36)		1.23 ± 0.05 (0.15)	3.44 ± 0.50 (0.46)	4.59 ± 2.68 (0.56)	0.54 ± 0.04
∑MUFA	**111.00 ± 6.83**^**e**^**(20.33)**	**150.97 ± 4.44**^**c,d**^**(20.54)**	**160.03 ± 0.50**^**b,c**^**(20.05)**	**146.72 ± 7.25**^**d**^**(19.59)**	**161.42 ± 1.67**^**b,c**^**(19.88)**	**174.37 ± 4.47**^**a**^**(21.11)**
C18:2n6t						
C18:2n6c	15.49 ± 0.59 (2.84)	23.94 ± 0.67 (3.26)	25.10 ± 0.63 (3.14)	25.65 ± 0.74 (3.42)	24.74 ± 0.69 (3.05)	21.74 ± 1.24 (2.63)
C18:3n6	1.51 ± 0.04 (0.28)	2.72 ± 0.19 (0.37)	2.52 ± 0.46 (0.31)	2.87 ± 0.38 (0.38)	3.32 ± 0.93 (0.41)	2.06 ± 0.39 (0.25)
C18:3n3	17.80 ± 0.01 (3.26)	28.21 ± 1.10 (3.84)	28.46 ± 0.66 (3.56)	24.78 ± 2.37 (3.31)	21.86 ± 1.16 (2.69)	19.97 ± 0.22 (2.42)
C18:4n3	35.26 ± 8.22 (6.46)	62.63 ± 0.39 (8.52)	52.47 ± 1.65 (6.57)	44.06 ± 1.81 (5.88)	30.92 ± 1.53 (3.81)	30.80 ± 0.77 (3.73)
C20:2						
C22:0 + 20:3n6	0.71 ± 0.11 (0.13)	1.32 ± 0.18 (0.18)	2.19 ± 0.50 (0.27)	1.94 ± 0.06 (0.26)	3.56 ± 1.21 (0.44)	1.48 ± 0.39 (0.18)
C20:3n3 + 22:1		0.90 ± 0.03 (0.12)	1.73 ± 0.33 (0.21)	1.94 ± 0.07 (0.26)	3.11 ± 1.21 (0.38)	1.16 ± 0.34 (0.14)
C20:4n6	12.40 ± 0.26 (2.27)	17.85 ± 0.29 (2.43)	17.36 ± 0.27 (2.17)	18.18 ± 1.06 (2.43)	26.98 ± 1.82 (3.32)	22.80 ± 1.63 (2.76)
C22:2						
C20:5n3	54.24 ± 2.10^f^(9.93)	74.34 ± 0.62^b,c^ (10.11)	78.82 ± 1.50^b^ (9.87)	60.85 ± 3.70^d,e,f^ (8.12)	73.67 ± 2.15^b,c^ (9.07)	68.81 ± 2.08^c,d^(8.33)
C22:5n3	2.24 ± 0.14 (0.41)		3.31 ± 0.11 (0.41)		12.98 ± 4.15 (1.60)	6.03 ± 0.17 (0.73)
C22:6n3	53.53 ± 4.21^e^(9.80 )	70.45 ± 0.41^d^(9.58)	91.0 ± 1.1^a^(11.52)	93.05 ± 5.50^a^(12.42)	97.98 ± 1.79^a^(12.06)	88.87 ± 6.84^a,b^ (10.76)
∑ PUFA	**193.183 ± 14.51**^**d,e**^**(35.38)**	**282.38 ± 1.90**^**a,b**^**(38.42)**	**300.67 ± 3.40**^**a**^**(38.03)**	**273.33 ± 13.31**^**a,b**^**(36.49)**	**299.14 ± 12.98**^**a**^**(36.84)**	**263.72 ± 13.74**^**b,c**^**(31.93)**

SAFA was the dominant group, followed by PUFA and MUFA, except in December when PUFA showed the highest content (293.8 mg/100 g) corresponding to 43% of total FAs Table 2. Significant variations occurred during the sampling period in SAFA content (ANOVA, *p* < 0.05) that ranged from 241.8 mg/100 g (corresponding to 44.3% of Total Fas) in March to 428.0 mg/100 g (50.5% of total Fas) in September. In all months, palmitic acid was clearly the predominant SAFA (C16:0: 161.6- 289.3 mg/100 g in October and September, respectively), followed by myristic (C14:0: 10.5–80.2 mg/100 g, in September and November, respectively) and stearic acids (C18:0), with a relative abundance of 94.7 in September 2014 and 67.0 mg/100 g in August 2015.

Significant differences in MUFA content (mg/100 g) were also observed during the study period, with minimum value in March (111.0 mg/100 g) corresponding to 20.3% of total FAs and maximum in September 2014 and August 2015 (175.7 and 174.4 mg/100 g, respectively) both corresponding to 21% of total FAs. Among them, palmitoleic (C16:1: 46–111.7 mg/100 g) and oleic acids (C18:1n9c: 36.2–100.6 mg/100 g) were dominant in all harvesting months (Table 2).

PUFA was the second most important group, ranging from 82.9 in November to 300.7 mg/100 g in May. The variation in the levels of PUFA was found to be significant among sampling months (ANOVA, *p* < 0.05). EPA and DHA were identified as the primary PUFAs, both FAs showed the lowest values in November (26.2 and 19.6 mg/100 g, respectively), and the highest values in December and spring-summer months for EPA and in late-spring and summer months for DHA (Table 2).

The nutritional quality of lipid observed in the species studied was evaluated by health lipids (Table 3). The highest contents of EPA + DHA were observed during May (170.8 mg/100 g) and summer months (153.7–171.6 mg/100 g) with another peak in December (166.6 mg/100 g) (Table 3).

**Table 3:** Seasonal variation of nutritional lipid indices in soft tissues of *Flexopecten glaber*. Lipid nutritional quality indices of the soft tissue of *Flexopecten glaber* reared in the Gulf of Taranto from September 2014 to August 2015. In the bracket are reported the % values of total Fatty acids. Values are mean ± S.D.(*N* = 30, *n* = 3). Different letters indicate significant differences for data in the same row (*p* < 0.05).

	September	October	November	December	January	February
EPA +DHA	139.27 ± 26.5^c^ (16.43)	104.55 ± 4.2^d^ (17.5)	45.89 ± 4.0^e^ (8.0)	166.57 ± 18.3^a^ (24.3)	106.29 ± 3.2^d^(17.3)	101.02 ± 0.5^d^(17.2)
DHA/EPA	1.40 ± 0.1^b^	0.54 ± 0.0^i^	0.75 ± 0.0^g^	0.77 ± 0.0^f,g^	0.65 ± 0.0^h^	0.83 ± 0.0^f^
∑ n3	186.19 ± 35.7^d^(22.0)	133.12 ± 2.2^f^(22.1)	58.76 ± 6.9^g^ (10.2)	236.10 ± 21.6 ^a,b,c^(34.4)	167.70 ± 5.6^d,e^ (27.2)	161.20 ± 6.0^e^ (27.4)
∑ n6	54.34 ± 45.7 ^a,b^(6.41)	34.28 ± 1.6^d^(5.7)	21.78 ± 1.9^e^ (3.8)	54.0 ± 11.2^a,b^ (7.9)	33.01 ± 0.8^d^ (5.4)	34.08 ± 0.9^d^ (5.8)
n3/n6	3.41 ± 0.4^f^	3.89 ± 0.1^e^	2.70 ± 0.1^g^	4.44 ± 0.5^c,d^	5.08 ± 0.1^a,b^	4.73 ± 0.0^b,c^
n6/n3	0.29 ± 0.0^b^	0.26 ± 0.0^c^	0.37 ± 0.0^a^	0.23 ± 0.0^d,c^	0.20 ± 0.0^f,g^	0.21 ± 0.0^e,f^
PUFA/SAFA	0.57 ± 0.1^f^	0.63 ± 0.0^e,f^	0.24 ± 0.0^g^	1.20 ± 0.3^a^	0.73 ± 0.0^c,d,e^	0.71 ± 0.0^c,d,e,f^
UNS/SAT	0.98 ± 0.1^e^	1.27 ± 0.0^c,d^	0.65 ± 0.1^f^	1.77 ± 0.2^a^	1.25 ± 0.1^c,d^	1.13 ± 0.0^d,c^
AI	0.81 ± 0.1^a,b^	1.20 ± 0.0^e^	2.41 ± 0.4^f^	0.58 ± 0.1^a^	1.12 ± 0.1^d,e^	1.16 ± 0.1^d,e^
IT	0.60 ± 0.1^e^	0.49 ± 0.0^d^	1.26 ± 0.1^f^	0.29 ± 0.0^a^	0.44 ± 0.0^b,c,d^	0.46 ± 0.0^c,d^
HH	1.07 ± 0.1^c,d,e^	0.83 ± 0.0^b^	0.38 ± 0.1^a^	1.51 ± 0.3^f^	0.87 ± 0.1^b^	0.91 ± 0.1^b,c^
	**March**	**April**	**May**	**June**	**July**	**August**
EPA+DHA	107.78 ± 6.3^d^(19.7)	144.79 ± 0.2^b,c^ (19.7)	170.83 ± 0.3^a^(21.4)	153.90 ± 9.2^a,b,c^(20.5)	171.65 ± 3.94^a^(21.1)	157.68 ± 8.9^a,b^ (19.1)
DHA/EPA	0.98 ± 0.0^e^	0.95 ± 0.0^e^	1.17 ± 0.0^d^	1.53 ± 0.0^a^	1.33 ± 0.0^c^	1.29 ± 0.1^c^
∑ n3	163.07 ± 14.7^d,e^(29.8)	236.54 ± 0.9^a,b,c^ (32.1)	253.49 ± 3.0^a^(32.1)	224.69 ± 13.3^b,c^(30.0)	240.52 ± 9.7^a,b^ (29.6)	215.63 ± 10.1^c^(26.1)
∑ n6	30.11 ± 0.2^d^(5.5)	45.84 ± 0.9^c^ (6.2)	47.17 ± 0.4^c^(5.9)	48.64 ± 0.0^b,c^(6.5)	58.61 ± 3.3^a^(7.2)	48.08 ± 0.3^b,c^(5.8)
n3/n6	5.42 ± 0.5^a^	5.16 ± 0.1^a,b^	5.37 ± 0.0^a^	4.62 ± 0.4^c^	4.10 ± 0.1^c,d^	4.49 ± 0.1^c,d^
n6/n3	0.18 ± 0.0^g^	0.19 ± 0.0^f,g^	0.18 ± 0.0^g^	0.22 ± 0.0^e,f^	0.24 ± 0.0^c,d^	0.22 ± 0.0^,e^
PUFA/SAFA	0.80 ± 0.1^b,c,d^	0.94 ± 0.0^b^	0.90 ± 0.0^b^	0.83 ± 0.0^b,c,d^	0.85 ± 0.1^b,c^	0.68 ± 0.0^d,e,f^
UNS/SAT	1.26 ± 0.1^c,d^	1.44 ± 0.0^b^	1.38 ± 0.0^b,c^	1.28 ± 0.0^b,c,d^	1.31 ± 0.1^b,c^	1.13 ± 0.0^d.,c^
AI	0.95 ± 0.1^b,c,d,e^	0.84 ± 0.1^b,c^	0.95 ± 0.0^b,c,d^	1.06 ± 0.2^c,d,e^	0.74 ± 0.1^a,b^	0.92 ± 0.0 ^b,c,d^
IT	0.41 ± 0.0^b,c,d,e^	0.35 ± 0.0^a,b^	0.36 ± 0.0^a,b,c^	0.40 ± 0.0^b,c,d^	0.39 ± 0.0^a,b,c,d^	0.47 ± 0.0^d^
HH	1.01 ± 0.1^b,c,d^	1.11 ± 0.1^d,e^	1.14 ± 0.0^d,e^	1.07 ± 0.1^c,d,e^	1.23 ± 0.1^e^	1.07 ± 0.0 ^c,d,e^

The amounts of n3 PUFA showed a decrease in autumn samples and an increase in December and spring-summer months. n6 PUFA also showed some variations, with highest levels in July, September and December (58.6, 54.3 and 54.0 mg/100 g, respectively) and lowest in November (21.8 mg/100 g) (ANOVA, *p* < 0.05) (Table 3). Among them, the most abundant were linoleic acid (C18:2n6), arachidonic acid (C20:4n6) (Table 2).

The n3/n6 ratio significantly varied during the sampling periods (ANOVA, *p* < 0.05), showing values below 4.0 in autumn samples.

PUFA/SAFA ratio significantly varied from the lowest value (0.24) in November to the highest (1.20) in December (ANOVA, *p* < 0.05) (Table 3).

The atherogenic (AI) and thrombogenic (TI) indexes exhibited significant changes over the study period (ANOVA, *p* < 0.05); the lowest values of AI and TI were found in December (0.58 and 0.29, respectively).

The highest values of HH were observed only in July and December (1.23 and 1.51, respectively), when also PUFA/SAFA ratios were high. The lowest HH levels were observed during autumn months, with 0.38 in November (Table 3).

**Figure 3:**
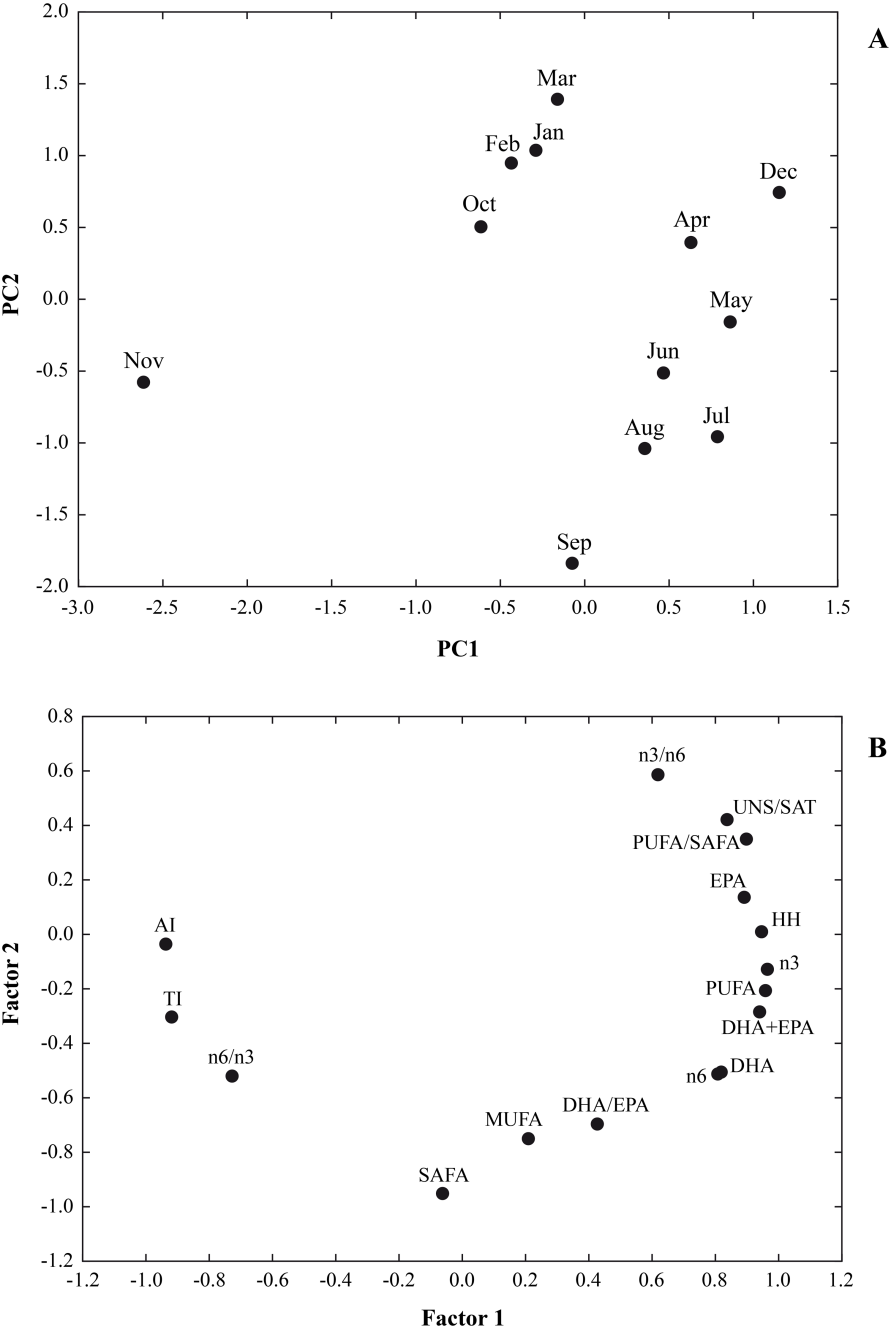
A multivariate analysis PCA was performed on a data set of 12 cases (12 months) and 16 variables (∑SAFA, ∑MUFA, ∑PUFA, n3, n6, n3/n6, n6/n3, PUFA/SAFA, UNS/SAT, EPA, DHA DHA/EP, DHA+EPA, AI, TI and HH). Principal components analysis (PCA) based on the lipid nutritional quality indexes of the *Flexopecten glaber* reared in the Gulf of Taranto from September 2014 to August 2015. (A) Scatterplot of PC2 against PC1; (B) Factor Loadings, Factor 1 vs. Factor 2.

In Fig. 3 the results of a multivariate analysis PCA was reported (Fig. 3). The combined plot of scores and loadings allowed us to recognize groups of samples with similar behaviour and the existing correlation among the original variables.

The values for the variables examined were 64.04% and 23.09% for PC1 and PC2, respectively. Therefore, the two-axis ordination, diagram described 87.13% of the variation. The scatter plot of scores on the first two principal components PC1 and PC2 shows a separation among the sampling time. Loading of variables on the first two principal components indicated that UNS/SAT, PUFA/SAFA, EPA, HH, n3, n6, DHA+EPA were characteristic component of biochemical composition of *F. glaber*, in December, May and July, while n6/n3, AI and TI with negative score on PC1, showing a lower beneficial effect in November.

Furthermore, the negative part of the PC2, SAFA, MUFA and DHA/EPA, corresponded to samples which contained higher quantities of these fatty acids in September, August and July and the lowest in March, February, January and December (Fig. 3).

## Discussion

Since molluscs represent an important part of the global seafood market and are well appreciated by consumers, numerous studies on monthly variations of their biochemical constituents have been carried out in many parts of the world, aiming to achieve an understanding of the nutritive value (e.g., Bongiorno et al., 2015; Berik, Çankiriligil & Gül, 2017a; Berik, Çankiriligil & Gül, 2017b).

The results of this study showed that the commercial traits (CI and MY), the proximate and fatty acids composition of *F. glaber*, reared in suspended cages, varied at different sampling times of the year.

Condition index and meat yield showed very similar patterns with peak values occurring in December, in spring months and in June and lowest values in mid-autumn. These findings are probably due to the major energetic expenditure required during the poor environmental seasons (Biandolino, Prato & Caroppo, 2008) for meat and gamete production. The results obtained are comparable to those collected in Lapseki Bay in Canakkale (Turkey) for the same species, which showed MY values in a range of 29.96% in summer and 39.69% in spring (Berik, Çankiriligil & Gül, 2017b). Similar results were also shown by mussels cultivated in the Gulf of Trieste, with 32.7% of MY in summer samples (Bongiorno et al., 2015), and in the same period in Galicia and Valencia (Spain), values of 31% and 34%, respectively (Fuentes et al., 2009). Moreover, several studies have demonstrated that the condition of bivalves and their biochemical composition is related also to environmental conditions, species, growth and food availability (Orban et al., 2002; Orban et al., 2006; Dridi, Salah Romdhane & Elcafsi, 2007; Biandolino, Prato & Caroppo, 2008; Berik, Çankiriligil & Gül, 2017b).

Marčeta et al. (2016) reported a resting gonad period, from October to December, for *F. glaber,* harvested in NW Adriatic Sea, with minimum values of GSI and two spawning periods: one mostly between July and September (with maximum values in June of 40%) and another minor spawning period in April–May. The GSI values recorded in this study were similar to that of *Pecten jacobaeus* and *Aequipecten opercularis* from the NW Adriatic Sea, that exhibit an appreciably longer spawning activity throughout the year with events of gamete emission during warm (spring-summer) and cold (winter-time) seasons (Castagnolo, 1991). Previous works reported GSI values of about 14.5% and 22% for *Argopecten irradians* from NW Atlantic (Barber & Blake, 1983); 22% (Sastry, 1979), 25% for *Aequipecten tehuelchus* from SW Atlantic (Narvarte & Kroeck, 2002) and 21% for *Patinopecten caurinus* from NE Pacific (MacDonald & Bourne, 1987).

These indices reflect the ecophysiological state of bivalves and their fluctuations have important implications for cultivation and harvesting strategy, since the commercial value of bivalves is reduced when they discharge their gonads during the spawning period. For optimum exploitation, the harvesting season should be timed to coincide with the peak period for these indexes (Okumus & Stirling, 1998).

The biochemical composition of many bivalves indicates annual patterns of both accumulation and use of reserves that is due to a complex interaction between food availability, growth and reproduction (Orban et al., 2002; Narváez et al., 2008).

Protein and lipid showed a similar pattern, with the highest values during spring-summer and the lowest in the autumn-winter months. This could be due to an accumulation of reserve substances, to be used during gamete ripening or to a greater muscle growth, indeed the increase of the GSI, CI and MY were correlated with lipid and protein content (GSI, *r* = 0.50 and *r* = 0.45, respectively; CI, *r* = 0.35 and *r* = 0.45, respectively and MY, *r* = 0.47 and *r* = 0.53, respectively; *t*-test, *p* < 0.05).

Carbohydrate showed a similar trend to that reported by Irisarri, Fernández-Reiriz & Labarta (2015) in *M. galloprovincialis* from Spain, which observed a reduced carbohydrate content in spring-summer and a recovered in autumn. The proximate composition in specimens from the Ionian Sea was similar to that reported for *F. glaber* from the Bizerte lagoon in the North east of Tunisia (Telahigue et al., 2010), while Berik & Çankiriligil (2013) reported highest values of protein and lipids, in samples collected in Lapseki Bay in Canakkale (Turkey).

Limited studies exist on the fatty acids composition of scallops from different areas of the Mediterranean Sea, including Italian waters. Moreover, these studies often show a single sampling time for a certain year and region, but no study covers monthly variation in the fatty acid profile of scallops (Telahigue et al., 2010). Therefore, the comparison with other scallops species is very difficult because of the lack of studies of the monthly fatty acid profile on whole soft tissues.

In this study, fatty acids profiles of *F. glaber* showed pronounced seasonal variations. The autumn specimens were characterized by a low percentage of PUFA and a proportional increase of SAFA, whilst MUFA remained constant. Conversely, in late spring-summer samples and in December, PUFA increased, whereas SAFA decreased. This switch can be linked to different factors, such as biological (spawning, starvation) or environmental (food quality and quantity change, climatic events disturbing feeding or metabolic processes).

It is known that suspension feeding bivalves have phytoplankton as the main component of their diet, that supply essential fatty acids. The quality and the quantity of this food source changes during the year (Biandolino, Prato & Caroppo, 2008). DHA predominated over EPA in late spring and summer samples, this could reflect an ingestion of Dinophyceae while the ratio DHA/EPA was reversed in favour of EPA during the other sampling months indicating a diatom- or flagellate-based diet (Lavaud et al., 2018).

DHA and EPA are considered to be among the major beneficial nutrients obtained from seafood consumption. The benefits of these fatty acids are recognized and current recommendations are of the order of 500 mg EPA + DHA per person per day (ISSFAL, 2004).

The consumption of foods that contain relatively high levels of n3 PUFA and small amounts of n6 PUFA is favourable to human health (Simopoulos, 2002; Simopoulos, 2003). Modern Western diets typically have ratios of n3 to n6 of 1 to 10 some as high as 1 to 30; the average ratio of n3 to n6 in the Western diet is 1:15–1:16.7. The optimal ratio is thought to be 1: 4 or higher (Simopoulos, 2002). Since the *n* − 3∕*n* − 6 ratio in this study was 3-5:1, it resulted highly favourable for consumer’s health, especially in December and in spring-summer months.

For *F. glaber* harvested in the Bizerte lagoon (Tunisian coast), Telahigue et al. (2010) reported n3 values of 13.3% in the gonad and of 26% of total FAs in the muscle, comparable to values from this study. Manthey-Karl et al. (2015) indicated that for wild king scallops *Pecten maximus* caught in autumn, from the Atlantic coasts of France and Norway, n3 values were higher (47.2 and 49.3% of total FAs, respectively) than our results, but there were comparable values of n6 (4.4 and 4.6% of total FAs, respectively).

It is widely accepted that a high n3/n6 fatty acid ratio is healthful, particularly with regard to reducing the risk of coronary heart diseases, plasma lipid levels and cancer risks (Kinsella, Lokesh & Stone, 1990) and it can been used as an index for comparing the nutritional values of shellfish.

*F. glaber* reared in the Mar Grande of Taranto, showed values higher than 1 (as recommended by Chow, 2008) and this could be attractive to consumers interested in low-fat food choices with potential health benefits.

In the Bizerte lagoon (Tunisia), *F. glaber* showed a n3/n6 ratio of 3.82 for digestive gland, 2.33 for gonad and 2.54 for muscle (Telahigue et al., 2013). In the Chilean scallop, *Argopecten purpuratus,* Caers et al. (1999) found the highest values for adductor muscle of 12.1, and for male and female gonads of 11.3 and 11.9, respectively. Manthey-Karl et al. (2015) reported n3/n6 ratios of about 11 for *P. maximus* from the Atlantic coasts. However, in others bivalves species, Dridi, Salah Romdhane & Elcafsi (2007) observed the lowest value of the n3/n6 ratio in the gonad of oyster *Crassostrea gigas* (3.17), while in the meat of the clam *Chamelea gallina*, this ratio varies between 4.28 and 10.8 (Orban et al., 2006). Dernekbasi et al. (2015) reported the lowest n3/n6 ratio for *M. galloprovincialis* (1.44–2.23) cultured in offshore long-line system in the Black Sea.

Another useful factor for assessing the nutritional quality of the lipid fraction of foods is the PUFA/SAFA ratio, which is considered to be a measure of the propensity of the diet to influence the incidence of coronary heart disease. Foods with PUFA/SAFA ratios below 0.45 have been considered undesirable for the human diet because of their potential to induce cholesterol increase in the blood (Department of Health and Social Security, 1984). In western diets the ratio is about 0.6 and it is suggested that increasing it to near 1.0 would be of great health benefit (McLennan & Abeywardena, 2005). In this study, PUFA/SAFA was higher than the recommended value, except for November, indicating the health benefit of this species.

The lowest values of AI and TI were found in December and the highest HH values in July and December are beneficial for consumers. To our knowledge, no data is available in the literature for lipid quality indices for molluscs from the Mediterranean coast. Czech, Grela & Ognik (2015) reported for frozen *Mytilus edulis*, AI, TI and HH values of 0.80, 0.33 and 1.71, and similar results were given by Merdzhanova et al. (2014) and Stratev et al. (2017) for *Mytilus galloprovincialis*.

## Conclusions

The value of this research was to fill gaps in knowledge of the commercial and nutritional characteristics of *F. glaber* present in the Italian market at various times of the year.

This information has important implications for consumers and farmers that have an interest to promoting a profitable scallop culture along Ionian coasts (Central Mediterranean Sea).

Their commercial and nutritional quality was generally good throughout most part of the year, although it seemed more valuable during spring-summer and December, mostly in term of total amount of PUFA, n3, DHA+EPA which attributed to health benefits for consumers. The smooth scallops is characterized by higher values of PUFA/SAFA and n3/n6 ratios than recommended level. The result would suggest that this scallop species is an effective source of essential fatty acids, especially DHA and EPA, showing that is a good food with potential health benefits in disease prevention. In conclusion, *F. glaber* can be proposed for a diversification of Italian shellfish culture that could also represent a new economical resource for farmers.

##  Supplemental Information

10.7717/peerj.5810/supp-1 Data S1 Raw data

## Additional Information and Declarations

### Data Availability

The following information was supplied regarding data availability: The raw data are provided as Data S1.
